# Myxovirus Resistance Protein 1 (MX1), a Novel HO-1 Interactor, Tilts the Balance of Endoplasmic Reticulum Stress towards Pro-Death Events in Prostate Cancer

**DOI:** 10.3390/biom10071005

**Published:** 2020-07-06

**Authors:** Emiliano Ortiz, Pablo Sanchis, Juan Bizzotto, Sofia Lage-Vickers, Estefania Labanca, Nora Navone, Javier Cotignola, Elba Vazquez, Geraldine Gueron

**Affiliations:** 1 Laboratorio de Inflamación y Cáncer, Departamento de Química Biológica, Facultad de Ciencias Exactas y Naturales, Universidad de Buenos Aires, Buenos Aires C1428EGA, Argentina; emilianogortiz@gmail.com (E.O.); pabloasanchis@gmail.com (P.S.); juanantoniobizzotto@gmail.com (J.B.); sofilage@gmail.com (S.L.-V.); jcotignola@qb.fcen.uba.ar (J.C.); elba@qb.fcen.uba.ar (E.V.); 2 CONICET-Universidad de Buenos Aires, Instituto de Química Biológica de la Facultad de Ciencias Exactas y Naturales (IQUIBICEN), Buenos Aires C1428EGA, Argentina; 3 Department of Genitourinary Medical Oncology and the David H. Koch Center for Applied Research of Genitourinary Cancers, The University of Texas MD Anderson Cancer Center, Houston, TX 77030, USA; estefania.labanca@gmail.com (E.L.); nnavone@mdanderson.org (N.N.)

**Keywords:** heme-oxygenase 1 (HO-1), myxovirus resistance protein (MX1), endoplasmic reticulum stress, unfolded protein response, prostate cancer

## Abstract

The inflammatory tumor microenvironment is a fertile niche accelerating prostate cancer (PCa). We have reported that heme-oxygenase (HO-1) had a strong anti-tumoral effect in PCa. We previously undertook an in-depth proteomics study to build the HO-1 interactome in PCa. In this work, we used a bioinformatics approach to address the biological significance of HO-1 interactors. Open-access PCa datasets were mined to address the clinical significance of the HO-1 interactome in human samples. HO-1 interactors were clustered into groups according to their expression profile in PCa patients. We focused on the myxovirus resistance gene (*MX1*) as: (1) it was significantly upregulated under HO-1 induction; (2) it was the most consistently downregulated gene in PCa vs. normal prostate; (3) its loss was associated with decreased relapse-free survival in PCa; and (4) there was a significant positive correlation between *MX1* and *HMOX1* in PCa patients. Further, *MX1* was upregulated in response to endoplasmic reticulum stress (ERS), and this stress triggered apoptosis and autophagy in PCa cells. Strikingly, *MX1* silencing reversed ERS. Altogether, we showcase MX1 as a novel HO-1 interactor and downstream target, associated with ERS in PCa and having a high impact in the clinical setting.

## 1. Introduction

The principal goal currently within prostate cancer (PCa) research is to unveil markers for the early detection of aggressive tumors that are predestined to invade and metastasize and to find druggable targets. Proteomics represents an important tool for the identification of new molecular targets for PCa-tailored therapy. Towards this end, we previously undertook an in-depth mass spectrometry-based study to build the heme-oxygenase 1 (HO-1) interactome in PCa. Heme-oxygenase 1, a critical mediator of cellular homeostasis [1], appears as an interesting target in oncology [2]. Although different roles have been attributed to HO-1 and tumor pathology [3], we have shown its anti-tumoral effects in PCa. This factor was capable of inhibiting cell proliferation, migration, and invasion [4]; it impaired tumor growth and angiogenesis in vivo and downregulated target genes associated with inflammation [4,5]. Interestingly, we have also found a tight association between HO-1 and the cytoskeletal compartment, altering adherens junctions and cell-cell zippering in PCa [6,7,8]. We have established a four molecular pathway foundation (ANXA2/HMGA1/POU3F1; NFRSF13/GSN; TMOD3/RAI14/VWF; PLAT/PLAU) behind HO-1 regulation of the tumor cytoskeletal compartments [8].

We propose that HO-1 and its interactors reprogram PCa cells and, in turn, modify the tumor microenvironment, favoring a less aggressive phenotype. Thus, we were interested in addressing the clinical significance of HO-1 interactors in PCa. In particular, as HO-1 is a stress response protein [9], we sought interactors associated with cellular stress.

The constant division of tumor cells represents a challenge given by the restriction in nutrients and oxygen supplies, supporting the selection of cell variants. This characteristic represents one of the reasons why proteins display altered expression patterns in cancer.

The functional complexity of the endoplasmic reticulum (ER) can be affected by a number of factors, both from the cell interior itself and/or from the environment that surrounds it, causing ER stress (ERS) [10]. Such factors include hypoxia, glucose availability, hypernatremia, redox status, and calcium levels, which directly impact the correct folding of proteins within the endoplasmic lumen, thus generating an accumulation of unfolded or misfolded proteins within ER [10]. This event triggers a specific cellular process in order to reestablish cellular proteostasis, which is known as the response to misfolded proteins (unfolded protein response (UPR)) [11]. Activation of this mechanism is the criterion that defines ERS [10]. The suboptimal conditions of the tumor microenvironment are the main triggers that make UPR activation a vital mechanism for the survival of tumor cells. Several observations have shown that an aggravated increase in acute stress, as well as the extension in time of the stressful stimulus represent decisive factors when determining cell destiny regarding death or survival [12,13].

To explore the contribution of HO-1 interactors associated with ER stress in PCa, we first performed a thorough bioinformatics analysis to address the role of HO-1 interacting proteins in this disease. Proteins were clustered into three groups according to their expression profile. In parallel, we carried out an RNA-seq analysis to compare gene expression profiles between PCa cells over-expressing HO-1 and their respective controls, to evaluate the ability of HO-1 in regulating the expression at the transcriptional level of the proteins included in these clusters. 

Taking into account all of our results, we focused on the human myxovirus resistance gene (*MX1*). This factor is well described as playing a vital role in preventing the replication of various types of RNA viruses [14,15,16]. Interestingly, some reports demonstrated that cells infected with the Influenza virus increased apoptosis mediated by ERS [14]. Hence, we sought to assess whether there was an association between *MX1* and ERS in PCa and, in turn, whether this association was linked to pro-apoptotic or pro-autophagic events. Further, we analyzed *HMOX1*, *MX1*, and ERS as risk predictors of clinical outcome in PCa.

## 2. Materials and Methods 

### 2.1. Cell Culture, Treatments, Reagents, and Antibodies

PC3 cells were obtained from the American Type Culture Collection (Manassas, VA, USA) and cultured with RPMI 1640 culture medium (Invitrogen, Carlsbad, CA, USA) supplemented with 10% *v*/*v* fetal bovine serum (FBS) (Internegocios, Buenos Aires, Argentina), penicillin 100 U/mL, streptomycin 100 µg/mL, and amphotericin 0.5 µg/mL. Hemin was obtained from SIGMA-Aldrich (St. Louis, MO, USA). For treatments, cells were incubated 24 h in RPMI media containing 10% FBS and antibiotics and then were exposed to hemin (80 µM, 24 h). Thapsigargin was obtained from Sigma-Aldrich and dissolved in dimethyl sulfoxide (DMSO) and phosphate-buffered saline (PBS). For treatments, cells were incubated in complete RPMI media and treated with thapsigargin 0.1 µM or 0.25 µM for 24 h. Interferon gamma (INFγ) protein was obtained from ImmunoTools (Friesoythe, Germany). PC3 cells were treated with INFγ, 500 U/mL, for 18 or 24 h. 

Mouse anti-human HO-1 monoclonal antibody and rabbit anti-MX1 antibody were obtained from Abcam (Cambridge, UK). Mouse anti-human β-actin antibody, rabbit anti-LC3, and rabbit anti-GADPH antibodies were obtained from Cell Signaling (Danvers, MA, USA). Horseradish peroxidase (HRP) conjugated anti-mouse secondary antibody was obtained from Cell Signaling. Secondary antibodies conjugated to Alexa 555 and Alexa 647 fluorophores were obtained from Molecular Probes, Invitrogen.

### 2.2. Plasmids and Transient Transfections

PC3 cells were transiently transfected for 48 h with HO-1 expression plasmids (p3xFLAGHO-1 or pcDNA3HO-1) or empty vectors as controls (p3xFLAG or pcDNA3), as previously described [8]. Cells cultured in 10 cm plates were transfected using 10 µg of plasmid and 20 µL of polyethylene glycol (PEI) in a final volume of 200 µL of RPMI 1640 culture medium. Transfections were performed on plates with 3 mL of RPMI 1640 without FBS or antibiotics. Five hours post-transfection, the culture medium was replaced by RPMI 1640 complete with 10% *v*/*v* FBS and antibiotics in the previously mentioned concentrations.

### 2.3. RNA Isolation, c-DNA Synthesis, and Quantitative Real-Time Polymerase Chain Reaction

Total RNA was isolated with Quick-Zol (Kalium technologies, Buenos Aires, Argentina) according to the manufacturer’s protocol. cDNAs were synthesized with the RevertAid Premium First Strand cDNA Synthesis Kit (Fermentas, Waltham, MA, USA) and used for real-time PCR amplification with Taq DNA Polymerase (Invitrogen, Waltham, MA, USA) in a QuantStudio 3 Real-Time PCR System (Thermo Fisher Scientific, Waltham, MA, USA). *PPIA* was used as an internal reference gene. The data obtained were analyzed using the method of 2^-ΔΔCT^ [17]. Primers used for each gene were as follows (5′-3′): *MX1* Fw: AGGACCATCGGAATCTTGAC, Rv: TCAGGTGGAACACGAGGTTC; *HSPA5* Fw: ACCGCTGAGGCTTATTTGGGA, Rv: CGTCTTTGGTTGCTTGGCGT; *XBP1* Fw: TGGATGCCCTGGTTGCTGAA, Rv: GCACCTGCTGCGGACTCA; *DDIT3* Fw: GCAGCGACAGAGCCAAAATC, Rv: GCTTTCAGGTGGTGATGTATG; *PPIA* Fw: GGTATAAAAGGGGCGGGAGG, Rv: CTGCAAACAGCTCAAAGGAGAC.

### 2.4. RNA Sequencing

RNA-seq was performed as previously described [8]. 

### 2.5. Immunofluorescence Assay

Cells were seeded in 12 well plates at a density of 1 × 10^5^ cells per well on coverslips overnight. After cells were transiently transfected with pcDNA3HO-1 or empty vector, cells were fixed in ice-cold methanol for 20 min at 4 °C and permeabilized with 0.1% Triton X (*v*/*v*) in PBS for 5 min at room temperature. After washing twice with PBS, cells were blocked with 3% (*m*/*v*) bovine serum albumin (BSA)/PBS for 1 h at room temperature. Cells were incubated overnight at 4 °C with anti-MX1 and HO-1 antibodies diluted 1:200 in 3% (*m*/*v*) BSA/PBS. Negative controls were carried out using PBS instead of primary antibodies. Cells were washed with PBS and incubated with fluorescent secondary antibodies for 1 h at room temperature, then stained with DAPI, and imaged by confocal laser scanning microscopy, performed with an Olympus Fluo View FV 1000 microscope, using an Olympus 60X/1.20 NA UPLAN APO water immersion objective.

### 2.6. Protein Extraction, Sodium Dodecyl Sulphate-Polyacrylamide Gel Electrophoresis and Western Blot 

Total cell lysates and immunoblot analysis were carried out as previously described [18]. Briefly, cells were lysed with RIPA buffer (Tris HCl 50 mM pH 7.4; NaCl 150 mM; ethylenediaminetetraacetic acid (EDTA) 20 mM pH 8; sodium deoxycholate 1%; SDS 0.1%; Triton X-100 1%, 1 mM Na_3_VO_4_, 20 mM NaF, and 1 mM Na_4_ P_2_O_7_, pH 7.9) and homogenized. After 20 min of incubation at 4 °C, lysates were centrifuged at 12,000× *g* for 20 min at 4 °C and the supernatant kept at −80 °C. Lysates containing equal amounts of proteins (60 μg) were resolved on 7.5–12.5% Sodium Dodecyl Sulphate-Polyacrylamide Gel Electrophoresis (SDS–PAGE) depending on the molecular weight of the proteins under study. Page Ruler Plus Prestained Protein Ladder (Fermentas) was used for the estimation of molecular weight. Proteins were blotted to a Hybond-ECL nitrocellulose membrane (GE Healthcare, Little Chalfont, UK). Membranes were blocked with 5% dry non-fat milk in TBS containing 0.1% Tween 20 (TBST) for 1 h at room temperature and incubated with primary monoclonal antibodies diluted in TBST (overnight; at 4 °C). Membranes were then washed with TBST (10 min; three times) and incubated with horseradish peroxidase-labelled secondary antibody (1 h at room temperature).

### 2.7. Apoptosis Analysis by Flow Cytometry

The FITC Annexin V Apoptosis Detection Kit II (BD Pharmingen, San Jose, CA, USA) was used to assay thapsigargin-induced apoptosis in PC3 cells, according to the manufacturer’s instructions. Briefly, after treatment, cells were washed twice with cold 1× PBS and resuspended in binding buffer 1× at a concentration of 1 × 10^6^ cells/mL. From this suspension, one-hundred microliters were labeled with FITC Annexin V and propidium iodide (IP) and incubated for 15 min at room temperature in the dark. Finally, four-hundred microliters of binding buffer 1× were added and analyzed by flow cytometry (Attune ™ NxT Flow Cytometer, Thermo Fisher Scientific) in the corresponding channels (Ex/Em: 495 nm/519 nm and 543 nm/614 nm), and the results were analyzed with FlowJo 8.7 software. For the non-specific label control, between 5 and 15 µg of the purified recombinant Annexin V protein were added to the cell suspension and incubated at room temperature for 15 min before proceeding with the labeling step (FITC + IP).

### 2.8. siRNA Transfection

PC3 cells were transfected with ON-TARGETplus SMARTpool siRNA (Dharmacon; Pittsburgh, PA, USA, cat# L-011735-00-0005) to knock down *MX1* expression or with a non-specific sequence siRNA (siRNA scrambled) as a negative control (ON-TARGETplus siRNA, Dharmacon, cat# D-001810-01-05). An amount of 3 × 10^5^ PC3 cells was plated in six well multiwell plates until 50% of confluence. The culture medium in each plate was then replaced with 1.8 mL of complete RPMI 1640 medium and transfected with the corresponding siRNA in a concentration of 25 nM using Lipofectamine LTX (Invitrogen). Forty-eight hours later, thapsigargin 0.25 µM was added for 24 h until RNA extraction.

### 2.9. Statistical Analysis

The *t*-test or ANOVA with the Tukey post-test was used to ascertain statistical significance with a threshold of *p* < 0.05 (*), *p* < 0.01 (**), and *p* < 0.001 (***).

### 2.10. Bioinformatics Analysis

#### 2.10.1. Oncomine Meta-Analysis in Prostate Cancer Patients

We searched the public cancer microarray database Oncomine (http://www.oncomine.org) to identify expression microarrays that compared expressions in prostatic adenocarcinoma vs. prostate gland. To be included in our study, a dataset was required to (1) be generated from human prostate tumors and (2) compare prostate adenocarcinoma vs. normal prostate gland. Differential genes were considered when: (1) they presented a *p*-value < 0.05 and (2) had an increase or decrease in expression ≥ 1.5 times. Although the *p*-value criterion was strict for the dataset selection, some genes were considered even if the fold change or the gene rank was <1.5 or >10%, respectively, when the gene showed a significant over- or under-expression. Genes were ranked by their *p*-value for every analysis scoring a gene rank. The median rank was the median *p*-value rank across datasets, for each gene assessed.

Search/study selection: We performed a search for each HO-1 interactor protein. The resulting studies were analyzed on the basis of healthy prostate gland vs. prostate adenocarcinoma. The cited literature was reviewed to confirm that the analysis was as documented in the Oncomine database. All the selected papers are included in Table 1 of the Results Section.

#### 2.10.2. RNA-Seq Gene Expression and Correlation Analysis in Prostate Cancer Patients

For gene expression and correlation analysis, we used the dataset from the Prostate Adenocarcinoma Project of The Cancer Genome Atlas (TCGA-PRAD; http://cancergenome.nih.gov). This study includes RNA-seq data from 497 prostate tumor samples and normal adjacent to tumor tissue, measured by massively parallel sequencing (Illumina HiSeq). 

For transcriptomics data derived from normal tissues, we browsed the Genotype-Tissue Expression (GTEx) project (2013) (https://gtexportal.org/), a collection of 54 non-diseased tissues sites across 1000 healthy donors. For our study, we used RNA-seq gene expression data of prostate samples (*n* = 100).

#### 2.10.3. Gene Expression Microarray and Relapse-Free Survival in Prostate Cancer Patients

To study the impact of expression levels on relapse-free survival (RFS) for PCa patients, one dataset was selected according to the following criteria: (1) the study included gene expression and clinical data for each patient with ≥ 5 years of follow-up; (2) the study consisted of ≥60 samples; and (3) the study was published and available in public repositories.

We used the dataset from Ross-Adams 2015 (GSE70770) [19] GPL10558 series available in gene expression omnibus (GEO). It includes information of a prostate cancer patient cohort with 206 samples from men who had undergone radical prostatectomy and clinical follow-up of eight years, including relapse information (biochemical relapse). Biochemical relapse was defined according to the European Guidelines as a prostate specific antigen (PSA) persistent rise above 0.2 ng/mL). Tumor sample expression of 31,000 transcripts was measured by 47,000 probes using the Illumina HumanHT-12 V4.0 platform.

#### 2.10.4. cBioPortal Exome Analysis in Prostate Cancer Patients

We used cBioPortal (https://www.cbioportal.org/), an open source cancer genomics data platform, created by the Memorial Sloan-Kettering Cancer Center (MSKCC), to analyze the most common mutations, copy-number alterations, and gene expression of *MX1* and *HMOX1* in PCa patients (11 datasets, *n* = 3211 samples; last accessed April 2020). The criteria used in order to include datasets in our analysis were as follows: (1) type of cancer: Prostate adenocarcinoma or metastasis; (2) the study must be published; and (3) the study consists of a sample number > 60. 

#### 2.10.5. Statistical Analysis

Stata software (StataCorp LLC, College Station, TX, USA) was used to explore patients’ RFS, and survminer R package [20] was used to generate Kaplan–Meier (KM) curves. To find the cutoff value to stratify patients into two groups based on the gene expression levels, we used the minimal *p*-value approach from the Cutoff Finder tool [21]. For univariable analyses of prognostic factors, the log-rank test and Cox proportional hazard model regression were employed. The hazards ratio (HR) indicates the probability of relapse of a patient with high gene expression with respect to one with low gene expression, considering HR=1 for the latter case. Correlation analysis between *HMOX1* and *MX1* was performed using Graph Pad Software. The Pearson correlation coefficient was determined for all analyses. Statistical significance was set at *p* < 0.05. For copy-number alterations and gene expression analysis, ANOVA followed by Tukey post tests were performed to assess statistical significance with a threshold of *p* < 0.05 (*), *p* < 0.01 (**), and *p* < 0.001 (***).

## 3. Results

### 3.1. Clinical Relevance of the HO-1 Interactome in Prostate Cancer

**Figure 1:**
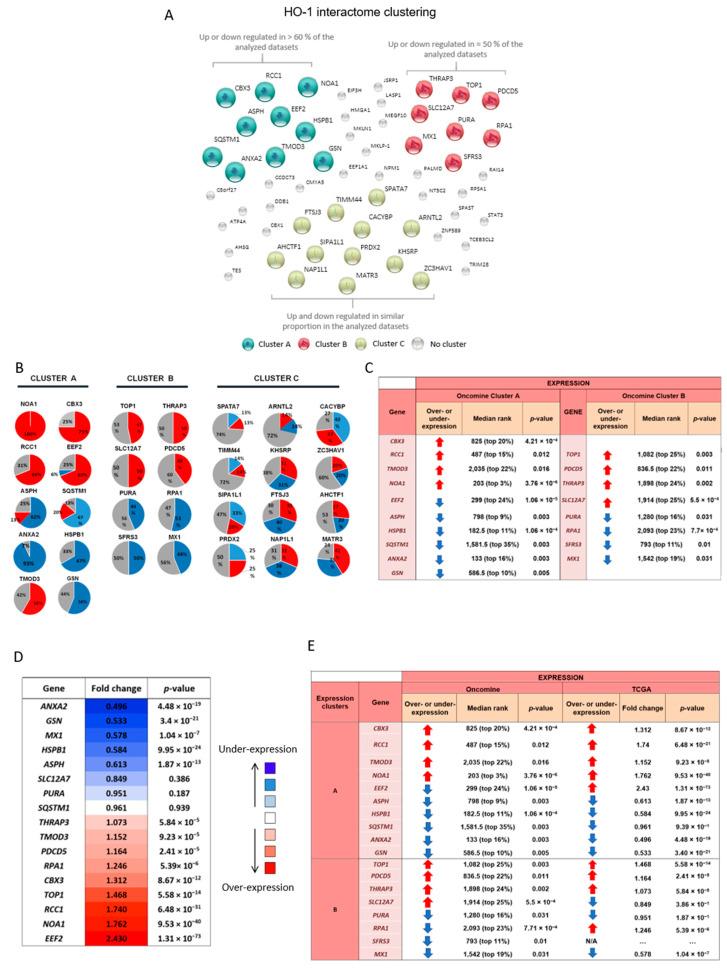
HO-1 interactome analysis. (**A**) HO-1 interactor proteins clustered according to their genetic expression profile in PCa patients in the Oncomine platform (*n* = 1045): Cluster A (aquamarine), Cluster B (red), Cluster C (yellow), and unclustered proteins (grey). Cluster A: genes that presented over- or under-expression in approximately 60% of the analyzed studies. Cluster B: genes that presented over- or under-expression in approximately 50% of the studies analyzed. Cluster C: genes expressed in similar proportions of over- or under-expression in the analyzed studies. (**B**) HO-1 interactor proteins were grouped according to their genetic expression profile. The graph shows the percentage of studies reporting gene under-expression (blue), over-expression (red), or without changes (gray) in each study analyzed in Oncomine (*n* = 1045). (**C**) Summary table showing the median rank and *p*-value for genes in Clusters A and B. (**D**) Gene expression analysis for genes in Clusters A and B comparing tumor vs. normal adjacent to tumor tissue, using the TCGA-Prostate Adenocarcinoma Project (PRAD) expression dataset (*n* = 497). The color code indicates no change in gene expression (white), over-expression (red), or under-expression (blue) for each gene. The intensity of the color is directly proportional to the gene’s expression fold change. The *p*-value for each gene is indicated in the third column. (**E**) The table summarizes the global analysis of the HO-1 interactome genes corresponding to Clusters A and B in Oncomine (16 datasets, *n* = 1045) and TCGA-PRAD (*n* = 497). The arrows indicate over- (red) or under-expression (blue) when comparing PCa vs. normal or normal adjacent to tumor tissue. Meta-analyses determined a median rank (position) for each gene assessed across all datasets. N/A = not available.

The molecular mechanisms underlying the pathogenesis of cancer-associated inflammation are intricate and represent a complex dialogue between the tumor and the microenvironment. As mentioned previously, HO-1 induction represents a critical event in cellular responses to pro-oxidative and pro-inflammatory insults [22]. HO-1 has been proposed to act as a biosensor regulating cell fate [23]. The current literature evidences that HO-1 has a role beyond its catalytic activity, participating in a wide variety of cellular processes that inhibit prostate tumor progression [1]. For this reason, we set out to identify HO-1-associated proteins and assess their clinical significance in PCa. We previously described the HO-1 interactome in PCa through a proteomics approach, identifying HO-1 molecular partners [8]. To address the clinical relevance of the HO-1 interactome in PCa, we searched the public cancer microarray database Oncomine (http://www.oncomine.org). This tool enabled us through pre-computed analyses to identify whether these genes had a high statistical significance for over- or under-expression in prostate adenocarcinoma vs. normal prostate gland. At the time of analysis, it harbored a total of 715 datasets and 86,763 human patient samples. We considered 16 datasets that met our eligibility criteria. Table 1 outlines the datasets selected, the total number of patients assessed (*n* = 1045), and the number of genes analyzed in each microarray. We elaborated a transcriptomic profile for each of the HO-1 interactors and classified them into three different clusters (A, B, or C) according to the following expression profiles (Figure 1A): Cluster A, genes that were over- or under-expressed in approximately 60% of datasets; Cluster B, genes that were over- or under-expressed in approximately 50% of datasets; Cluster C, genes that were over- or under-expressed in similar percentages of datasets (Figure 1A). Cluster A rendered the following genes: *NOA1*, *CBX3*, *RCC1*, *EEF2*, *ASPH*, *SQSTM1*, *TMOD3*, *GSN*, *HSPB1*, and *ANXA2* (Figure 1B). The pie charts represent the percentage of datasets where the gene under study was over-expressed in PCa vs. normal prostate (red) or under-expressed in the same comparison (blue) (Figure 1B). Cluster B included: *TOP1*, *PDCD5*, *THRAP1*, *SLC12A7*, *PURA*, *RPA1*, *SFRS3* and *MX1* (Figure 1B). Cluster C included: *ZC3HAV1*, *AHCTF1*, *TIMM44*, *KHSRP*, *MATR3*, *SIPA1L1*, *FTSJ3*, *NAP1L1*, *PRDX2*, *CACYBP*, and *ARNTL2* (Figure 1B). The meta-analysis combining data from the independent datasets assessed showed that for prostate adenocarcinoma vs. normal prostate gland, Cluster A and B genes lied within 35% of the most consistently high or low expressed genes across this comparison (Figure 1C). We also assessed all clusters’ expression profiles across The Human Protein Atlas platform (https://www.proteinatlas.org/). Although a few samples were available for prostate adenocarcinoma, we could observe a positive correlation for NOA, RCC1, TMOD3, HSPB1, ASPH, SQSTM1, and ANXA2 from Cluster A and TOP1, PDCD5, THRAP3, and MX1 from Cluster B (Supplementary Figure S1A).

In parallel, we examined gene expression for Clusters A and B using the data from the Prostate Adenocarcinoma Project of The Cancer Genome Atlas (TCGA-PRAD, *n =* 497) [24]. This dataset allowed us to evaluate gene expression profiles for PCa vs. normal adjacent to tumor tissue (Figure 1D). Values greater than one represent gene induction (red), while values lower than one represent gene downregulation (blue). Results showed a significant dysregulation of selected genes in tumor compared with normal adjacent to tumor tissues: *ANXA2* (fold change = 0.496; *p* = 4.48E-19), *GSN* (fold change = 0.533; *p* = 3.40 × 10^−21^), *MX1* (fold change = 0.578; *p* = 1.04 × 10^−7^), *HSPB1* (fold change = 0.584; *p* = 9.95 × 10^−24^), and *ASPH* (fold change = 0.613; *p* = 1.87 × 10^−13^); *EEF2* (fold change = 2.43; *p* = 1.31× 10^−73^), *NOA1* (fold change = 1.762; *p* = 9.53 × 10^−40^), and *RCC1* (fold change = 1.740; *p* = 6.48 × 10^−31^). Although the comparisons made in TCGA-PRAD and Oncomine were not exactly performed on the same tissues, some synchronicity could be observed between the expression profiles in both analyses for the genes studied (Figure 1E). Genes *CBX3*, *RCC1*, *TMOD3*, and *NOA1* (Cluster A) and *TOP1*, *PDCD5*, and *THRAP3* (Cluster B) were upregulated for both datasets in PCa tissue vs. normal gland or normal adjacent to tumor tissue. Genes *ASPH*, *HSPB1*, *SQSTM1*, *ANXA2*, and *GSN* (Cluster A) and *PURA* and *MX1* (Cluster B) were downregulated for both datasets in PCa tissue vs. normal gland or normal adjacent to tumor tissue (Figure 1F).

### 3.2. Transcriptomic Analysis of HO-1 Interactors and Risk of Relapse in Prostate Cancer Patients

To evaluate the relapse-free survival (RFS) time in PCa patients, associated with changes in the expression of Cluster A and B genes, we carried out an analysis using the Ross–Adams dataset (GSE70770) [19] (*n* = 206), which gathers expression and clinical data from PCa patients who had undergone radical prostatectomy. Kaplan–Meier (KM) curves were performed taking the low expression group as the control. Results showed that RFS was significantly lower in patients who had low expression of *TMOD3*, *EEF2*, *ASPH*, *HSPB1*, *SQSTM1*, *ANXA2*, *GSN*, *TOP1*, *THRAP3*, *SLC12A7*, *PURA*, and *MX1* or high expression of *RCC1*, *PDCD5*, *RPA1*, and *SFRS3* (Table 2 and Supplementary Figure S1B). When compiling all the expression and RFS data, we could sub-classify genes with high expression in PCa vs. normal gland or normal adjacent to tumor tissue and significantly decreased RFS (*RCC1* and *PCDC5*) or with low expression in PCa and decreased RFS (*ASPH*, *GSN*, *HSPB1*, *SQSTM1*, *ANXA2*, *MX1*, and *PURA*) (Table 2, white dots). In summary, the results evidenced how transcript dysregulation of these HO-1 interactors was associated with a change in RFS, highlighting their clinical importance for PCa.

### 3.3. mRNA Expression Profiles of HO-1 Interactors under Genetic Induction of HO-1

**Figure 2:**
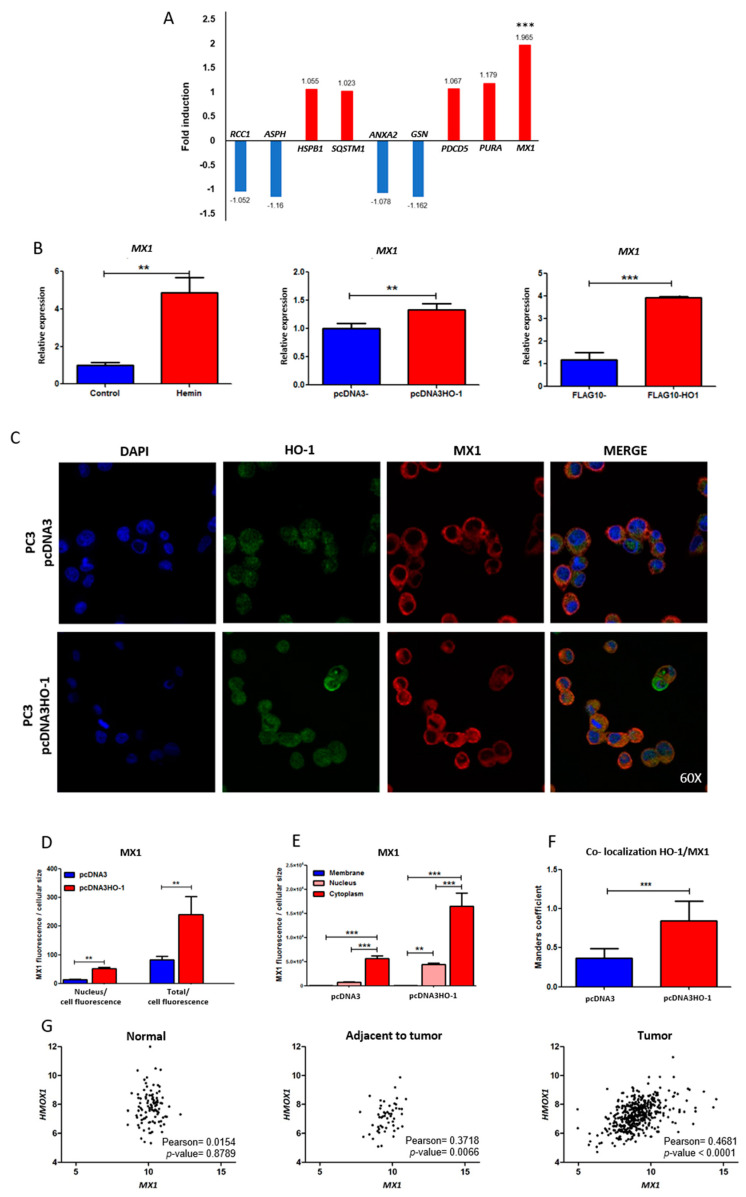
Effect of HO-1 modulation on the expression and localization of MX1. (**A**) RNA-seq expression for the selected genes in Table 2 under HO-1 induction. PC3 cells were transiently transfected with the pcDNA3HO-1 expression plasmid or empty vector. Forty-eight hours later, RNA was extracted, purified, and sequenced. (**B**) *MX1* assessed by real-time PCR (RT-qPCR) in PC3 cells treated with hemin (80 µM, 24 h) or PBS or transfected with the expression plasmids pcDNA3/pcDNA3HO-1 and FLAG/FLAGHO-1. The values were relativized using *PPIA* as a reference gene and normalized to the controls. (**C**) Immunofluorescence (IF) staining and confocal microscopy analysis (magnification: 60×) for HO-1 (green) and MX1 (red) on PC3 cells transfected with the expression plasmids pcDNA3 or pcDNA3HO-1. (**D**) Semi-quantitative analyses of the IF for MX1 by segmentation of the whole cell and nucleus (DAPI). The fluorescence intensity for MX1 and HO-1 was determined using ImageJ software and normalized to cell size (*n* ≥ 20 cells for each condition), and the ratios of nuclear label/total label and total fluorescence/cell were calculated. (**E**) MX1 staining in the membrane, cytoplasm, and nucleus. (**F**) Co-localization analysis of MX1 and HO-1 expressed as the Manders correlation coefficient. Error bars correspond to the standard deviation. (**G**) *HMOX1* and *MX1* Pearson correlation analysis in normal prostate (Genotype-Tissue Expression (GTEx) database, *n* = 100), normal adjacent to tumor, or prostate cancer tissues (TCGA-PRAD, *n* = 52 and *n* = 497, respectively). One representative of at least three independent experiments is shown. Results are shown as the mean ± S.D. Statistical significance: ** *p* < 0.01; *** *p* < 0.001.

In our previous work showing a comparative RNA-seq analysis between PC3 cells over-expressing HO-1 and controls [8], we found that HO-1 induction triggered the alteration of key cytoskeletal genes leading to a more adhesive and less invasive phenotype, further supporting its anti-tumoral function in PCa [8]. Here, we assessed whether HO-1 was capable of modulating the gene expression of the selected HO-1 interactors (Table 2, asterisks). Results showed that when PC3 cells were transiently transfected with the vector pcDNA3HO-1 or the empty vector as the control (pcDNA3), there was a significant change in the expression profile of *MX1* (Figure 2A). The rest of the genes assessed presented no significant alteration under HO-1 induction. Interestingly, while *MX1* appeared as downregulated in PCa compared to normal gland and was associated with poorer RFS (Figure 1 and Table 2), HO-1 modulation was capable of inducing its expression.

Thus, we decided to focus our attention on the *MX1* gene. First, we validated the RNA-seq analysis by real-time PCR (RT-qPCR). PC3 cells treated with hemin, a potent inducer of HO-1, showed a significant increase of *MX1* expression (fold induction: 4.88, *p* < 0.01). Further, genetic induction of HO-1 in PC3 cells, using two different expression vectors (pcDNA3HO-1 and FLAGHO-1), also caused a significant increase in *MX1* mRNA levels compared with cells transfected with empty vectors (fold induction: 1.33, *p* < 0.01 and 3.91, *p* < 0.001, respectively) (Figure 2B).

### 3.4. Effect of HO-1 Modulation on the Expression and Localization of MX1

Subsequently, in order to assess whether the increase in *MX1* was also reflected at the protein level and whether it showed changes in its subcellular localization, we performed an immunofluorescence analysis by confocal microscopy, using specific anti-MX1 and anti-HO-1 antibodies (Figure 2C). Results showed that induction of HO-1 in prostate tumor cells caused a significant increase in MX1 protein levels, observed by an enhancement in the total nuclei fluorescence and in the total cell fluorescence compared with cells transfected with empty vector (Figure 2C–E). Furthermore, we were able to determine by the Manders coefficient that there was a greater co-localization of HO-1 and MX1 when cells over-expressed HO-1 (pcDNA3HO-1) compared with controls (Figure 2F, *p* < 0.001). Altogether, HO-1 over-expression in PC3 cells triggered an increase in MX1 mRNA and protein levels and altered its subcellular localization, reflecting a clear association between both proteins.

### 3.5. Correlation of Expression between MX1 and HMOX1 Genes in Normal Prostate and Prostate Cancer Samples

We next assessed whether there was any correlation between *MX1* and *HMOX1* in tissues from healthy donors and PCa patients. Using the TCGA-PRAD database, we observed a positive correlation in the expression profiles of *MX1* and *HMOX1*, both in normal adjacent to tumor (Pearson = 0.3718; *p* = 0.0066) and in tumor tissue samples (Pearson = 0.4681; *p* < 0.0001) (Figure 2G). The *MX1* and *HMOX1* correlation was not detected in normal tissue. This evidenced a potential association between both genes in PCa.

### 3.6. Exome and RNA-Seq Correlation Analyses of MX1 and HMOX1 in Prostate Cancer

**Figure 3:**
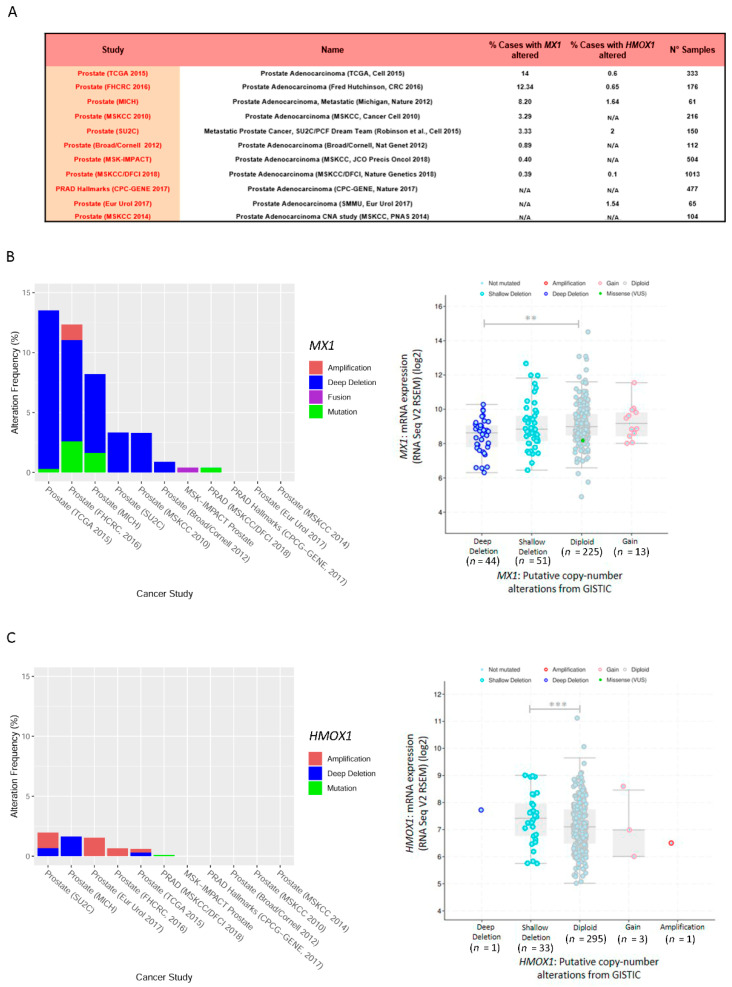
*MX1* and *HMOX1* exome and RNA-seq analysis in PCa patients. (**A**) Selected studies from cBioPortal for the genetic alterations’ analysis in *MX1* and *HMOX1* in PCa patients. The table shows the number of samples and the number of patients with alterations in target genes. (**B**,**C**) (left panels): Percentage of cases with alterations and the type of alteration observed: Point mutations (green), amplifications (red), fusion (purple), or complete deletions (blue) for *MX1* (**B**) and *HMOX1* (**C**), through 11 selected dataset (x-axis). Right panels: correlation between gene expression and copy number alterations for *MX1* (**B**) and *HMOX1* (**C**) in TCGA-PRAD (*n* = 333). Statistical significance: ** *p* < 0.01; *** *p* < 0.001. N/A = not available.

We extended the bioinformatics analysis, using cBioPortal for Cancer Genomics (https://www.cbioportal.org). Eleven datasets were selected that met our eligibility criteria (Figure 3A). We assessed whole exome and RNA-seq data to evaluate *MX1* and *HMOX1*. This analysis performed on PCa patient samples (*n* = 3211), showed that the most frequent gene alteration of *MX1* was deletion (Figure 3B, left panel). It should be noted that for the TCGA-PRAD dataset, such an alteration reached a frequency of 13.21% (Figure 3B, left panel). Next, we analyzed whether there was a correlation between *MX1* gene alterations and its expression profile. Results showed that for the TCGA-PRAD dataset, those samples with a complete *MX1* deletion presented lower mRNA expression, compared with the unaltered samples (diploid) (Figure 3B, right panel). The same analysis was carried out for *HMOX1*, and the results showed that the most common alterations were amplification and deep deletion (Figure 3C, left panel). However, the frequency of alterations in PCa patients was substantially lower (0.6%) compared with *MX1* (14%) (Figure 3B,C). Additionally, while most of the samples presented the two copies of *HMOX1*, only a smaller proportion of the samples showed superficial deletion (shallow deletion) without it being associated with a lower level of mRNA expression (Figure 3C, right panel). In summary, in silico analysis showed that PCa patients with *MX1* deletion correlated with lower *MX1* mRNA levels. 

### 3.7. MX1 and Endoplasmic Reticulum Stress in Prostate Cancer

Taking into account the results observed so far, the next step was to explore in PCa the possible functional role of *MX1*, the main IFN-inducible gene, and its association with ERS and with ERS-mediated apoptosis.

To address this goal, we generated ERS conditions in the tumor cells using a specific ERS inducer, thapsigargin. This drug inhibits the proper functioning of the sarco-/endo-plasmic reticulum Ca^++^-ATPase. This inhibition generates an imbalance in the Ca^2+^ ion homeostasis, which triggers ERS signaling [25]. In this work, we studied the expression of X-box binding protein 1 (*XBP1*), which participates downstream of the IRE1 activator; DNA damage inducible transcript 3 (*DDIT3*), a gene coding for the protein inducible by DNA damage and growth arrest (CHOP); and heat shock protein family A (Hsp70) member 5 (*HSPA5*), a gene encoding for BiP. This chaperone is the main activator of the ERS pathway [26]; therefore, an increase in *HSPA5* levels is considered a strong ERS marker [10].

**Figure 4:**
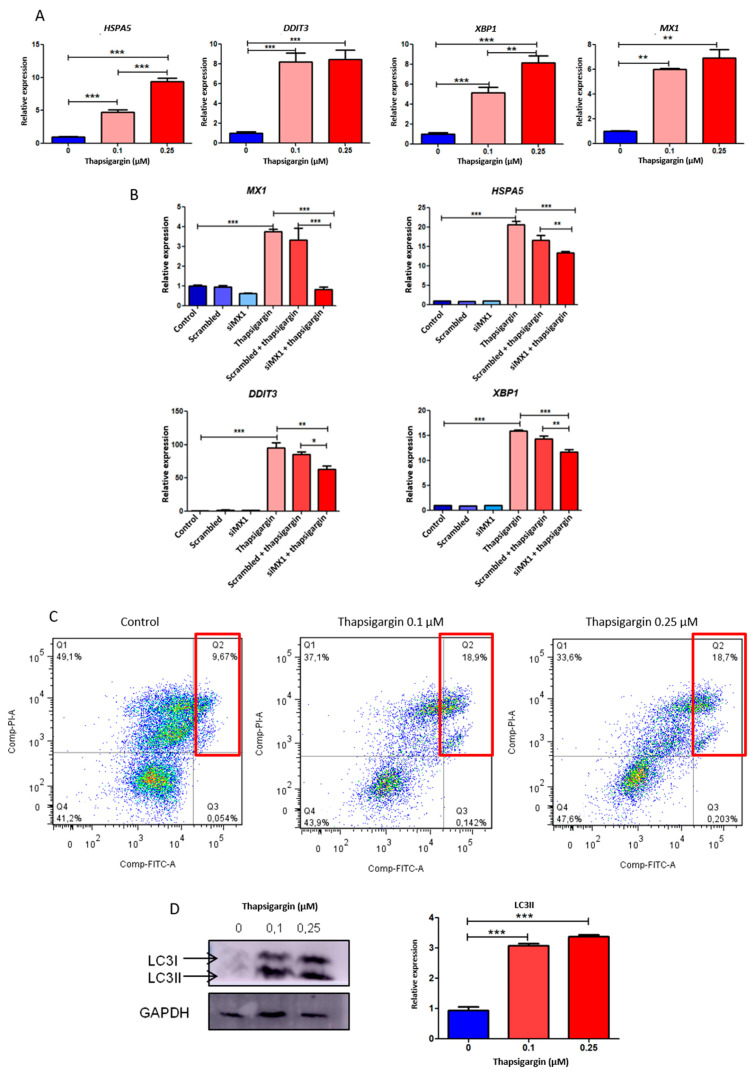
Implications of *MX1* and endoplasmic reticulum stress (ERS)-associated genes in PCa. (**A**) *HSPA5*, *DDIT3*, *XBP1*, and *MX1* expression assessed by RT-qPCR in PC3 cells treated with thapsigargin (0.1 and 0.25 µM; 24 h) or PBS as the control. Values were relativized using *PPIA* as a reference gene and normalized to the control. (**B**) *MX1*, *HSPA5*, *DDIT3*, and *XBP1* expression levels assessed by RT-qPCR in PC3 cells transfected with a *MX1*-targeted siRNA or a scrambled siRNA for 72 h and treated with 0.25 µM thapsigargin during the last 24 h of silencing. Values were relativized to *PPIA* as a reference gene and normalized to the control. (**C**) Flow cytometry graphs showing cell viability and apoptosis populations in PC3 cells treated with thapsigargin (0.1 and 0.25 µM; 24 h) or PBS as the control. Cells were treated with FITC-labeled Annexin V and PI staining. X axis: FITC channel; Y axis: PI channel. (**D**) LC3 I and LC3 II protein levels assessed by Western blot in PC3 cells treated with thapsigargin (0.1 and 0.25 µM; 24 h) or PBS as the control. Protein bands were quantified using ImageJ 1.52a software (NIH), normalized to GAPDH as a reference protein, and relativized to the control (right panel). One representative of at least three independent experiments is shown. Results are shown as the mean ± S.D. Statistical significance: * *p* < 0.05; ** *p* < 0.01, *** *p* < 0.001.

PC3 cells treated with thapsigargin showed a significant increase in *HSPA5* mRNA expression compared with the control (fold induction: 4.71, *p* < 0.001 and 9.41, *p* < 0.001, for 0.1 µM and 0.25 µM, respectively) (Figure 4A). Similarly, we could see a significant increase in mRNA levels of the UPR intermediate genes, *DDIT3* (fold induction: 8.21, *p* < 0.001 and 8.46, *p* < 0.001, 0.1 µM and 0.25 µM, respectively) and *XBP1* (fold induction: 5.17, *p* < 0.001 and 8.13, *p* < 0.001, 0.1 µM or 0.25 µM, respectively) (Figure 4A). These results demonstrated that ERS was triggered in PC3 cells in a thapsigargin concentration-dependent manner. We also assessed *MX1* expression under ERS induction. Results showed increased *MX1* mRNA levels compared with controls under thapsigargin treatment (fold induction: six, *p* < 0.01 and 7.4, *p* < 0.01, 0.1 µM or 0.25 µM, respectively) (Figure 4A). In summary, we reported *MX1* induction under ERS conditions, something not previously described in PCa.

We also confirmed *MX1* induction under IFNγ treatment (fold induction: 16.79, *p* < 0.001) (Supplementary Figure S2A). Since thapsigargin not only triggered an increase in ERS, but also generated an induction of *MX1* expression in PC3 cells, this led us to hypothesize if those conditions that raised *MX1* levels, such as hemin or IFNγ, were capable of inducing the expression of ERS genes. The results showed that IFNγ treatment caused a significant increase in *DDIT3*, *XBP1*, and *HSPA5* expression (Supplementary Figure S2B). On the other hand, when treating PC3 cells with hemin, a significant decrease in *DDIT3* and *HSPA5* expression was observed compared with controls. No change in *XBP1* expression was observed (Supplementary Figure S2C). These results indicated that hemin reduced the basal levels of ERS, contributing to reverse the state of cellular ERS.

Further, we knocked down *MX1* using *MX1* small interfering RNA (siMX1). As expected, thapsigargin-induced levels of *MX1* were significantly decreased by siMX1 (75%, *p* < 0.001) (Figure 4B). Moreover, a significant decrease by siMX1 was also observed for *HSPA5* (19.64%, *p* < 0.01), *DDIT3* (26.93%, *p* < 0.05), and *XBP1* (24.19%, *p* < 0.01), relativized to the transfection control (thapsigargin + scrambled) (Figure 4B). In summary, these results showed that *MX1* could regulate these genes under ERS conditions.

### 3.8. Effect of Thapsigargin-Induced Endoplasmic Reticulum Stress in Apoptosis and Autophagy in Prostate Cancer

We then evaluated whether ERS induction affected cellular processes such as apoptosis and autophagy. For this purpose, we performed flow cytometry analysis (Annexin V-FITC/IP) on PC3 cells, exposed to thapsigargin. The results clearly showed that this compound was able to trigger a significant increase in apoptosis compared with the control (Figure 4C). As observed earlier, cells grown in the presence of thapsigargin showed high levels of ERS and *MX1* induction.

Faced with a stimulus that triggers ERS, cells seek to reestablish homeostasis through the UPR signaling pathway. Autophagy is considered one of the highly characterized effectors of the UPR pathways. For this reason, we also analyzed the implication of this cellular process under ERS conditions in PCa. We assessed LC3 lipidation, indicated by the conversion of LC3-I into LC3-II. The results showed that endogenous levels of LC3-II accumulated upon thapsigargin treatment compared with controls (Figure 4D). These results supported the ideas that propose autophagy as an effector pathway of UPR once ERS is triggered in the cell.

### 3.9. Analysis of HMOX1, MX1, and Endoplasmic Reticulum Stress Genes as Risk Predictors of Clinical Outcome in Prostate Cancer

**Figure 5:**
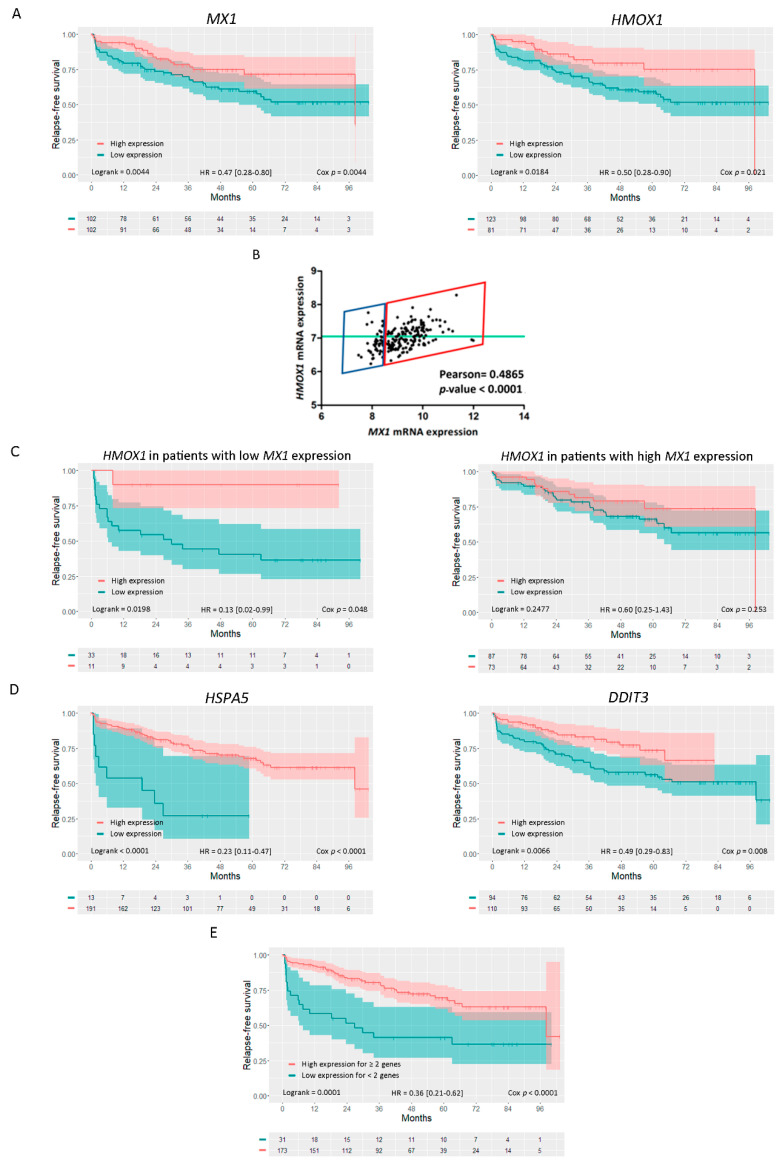
RFS and correlation analysis for *MX1*, *HMOX1*, and ERS related genes in PCa patients (Ross–Adams, GSE70770). (**A**) Kaplan–Meier (KM) curves for RFS in months for PCa patients with low (blue) or high (red) *MX1* (left panel) or *HMOX1* (right panel) expressions. (**B**) Gene expression correlation analysis between *HMOX1* and *MX1*, expressed as Pearson’s coefficient. (**C**) KM curves for RFS for PCa patients with high (red) or low (blue) expression of *HMOX1* segregated based on low (left panel) or high (right panel) *MX1* levels. (**D**) KM curves for RFS for PCa patients with high (red) or low (blue) expression of *HSPA5* (left panel) or *DDIT3* (right panel). (**E**) KM curve for the co-occurrent expression of *MX1*, *HMOX1*, *HSPA5*, or *DDIT3* in patients with two or more (red) or one/none (blue) highly expressed genes. All comparisons considered low expression patients as the reference group. HR: hazards ratio. Statistical significance: *p* < 0.05.

We next analyzed whether the alteration in *HMOX1*, *MX1*, and ERS genes’ expression could influence PCa patients’ RFS. Kaplan-Meier curves for the Ross–Adams dataset (GSE70770, *n* = 206) showed that higher expression levels for both *MX1* and *HMOX1* were associated with a better RFS in PCa patients (HR = 0.47, *p* = 0.0044 for *MX1* and HR = 0.50, *p* = 0.021 for *HMOX1*) (Figure 5A).

Next, we assessed the association between the expression profiles of *HMOX1* and *MX1* in the same dataset. In accordance with the TCGA-PRAD dataset (Figure 2G), we observed a positive correlation between *HMOX1* and *MX1* expression (Pearson = 0.4865, *p* < 0.0001) (Figure 5B).

Next, we categorized PCa patients based on *MX1* expression and performed KM curves to evaluate the effect of *HMOX1* in RFS of these patient subgroups (low or high *MX1* expression). Interestingly, while it was observed that patients with low *MX1* had a higher risk of biochemical relapse (Figure 5A), this risk decreased significantly in those patients who exhibited higher levels of *HMOX1* (HR = 0.13; *p* = 0.048) (Figure 5C, left panel). In contrast, the expression profile of *HMOX1* did not seem to influence the risk of biochemical relapse in those patients with high levels of *MX1* (HR = 0.6; *p* = 0.253) (Figure 5C, right panel). We then expanded the analysis for *HSPA5* and *DDIT3* and found similar results to those observed for *MX1* and *HMOX1*. Patients with higher expression for ERS genes showed a significantly lower risk of relapse than patients with lower expression for these genes (Figure 5D; HR = 0.23, *p* < 0.0001 for *HSPA5* and HR = 0.49, *p* = 0.008 for *DDIT3*). We also evaluated the effect of co-occurrent high expression for all genes. It is interesting to note that patients with high expression of two or more factors (*MX1*, *HMOX1*, *HSPA5*, or *DDIT3*) had a lower risk of biochemical relapse than patients with one or none of the highly expressed genes (HR = 0.36; *p* < 0.001) (Figure 5E).

These results suggested that higher levels of *MX1*, *HMOX1*, *DDIT3*, and *HSPA5* resulted in a better prognosis for PCa patients that have undergone radical prostatectomy.

## 4. Discussion

The induction of HO-1 represents an essential event in cellular responses to pro-oxidative and pro-inflammatory insults maintaining cellular homeostasis [1]. Thus, HO-1 has been proposed to act as a biosensor regulating cell destination [27]. Our previous reports have documented the nuclear expression of HO-1 in human primary prostate carcinomas naive of treatment [28]. We also showed that HO-1 is further implicated in PCa, demonstrating that endogenous HO-1 inhibits bone-derived prostate cancer cell migration, invasion, and proliferation [4] and negatively modulates the expression of pro-inflammatory and pro-angiogenic genes. Further, using a fully immunocompetent murine model, our reports revealed how stromal conditioning with hemin impaired PCa development by targeting both the tumor vasculature and the cytotoxic T-cell immune response [29]. These data, together with the reported bibliography, clearly suggest that HO-1 fulfills a key molecular and cellular function, beyond its enzymatic function.

It seems as if HO-1 could suppress the sustained inflammation in PCa, halting tumor progression [4,5]. However, it is logical to think that this anti-tumoral action directed by HO-1 is associated with other factors capable of interacting with HO-1 to carry out these functions. Hence, we sought to analyze the HO-1 interactome in PCa to unravel some of the molecular mechanisms underlying its anti-tumoral role and the acquisition of a less aggressive phenotype in PCa. In our previous work, we optimized the purifying technique to isolate HO-1 interacting proteins in PCa cells and build the HO-1 interactome. This provided a framework for identifying novel partners that could reprogram PCa cells, favoring the less aggressive tumor phenotype observed under HO-1 forced expression [4,5,8,30].

Although we studied prostate tumor tissues at the transcriptomic level, proteins involved in tumor evolution have been less explored in PCa. Different groups of genes, proteins, and metabolites are responsible for the progression from a precursor lesion to a localized disease and finally leading to a metastatic stage. To understand the clinical-pathological importance of proteins associated with HO-1, we carried out an in-depth bioinformatics analysis (Oncomine, n = 1045) using public datasets that allowed us to assess their clinical relevance in the disease. HO-1 interactor proteins were analyzed, and an expression profile was elaborated for each one of them. We clustered interactors according to their mRNA expression profiles in three groups, highlighting those genes that were over- or under-expressed in approximately >60% (Cluster A) or 50% (Cluster B) of all datasets in prostate adenocarcinoma vs. normal prostate gland and lied within 35% of the most consistently high or low expressed genes across this comparison. In parallel, we studied the expression of HO-1 interactors in TCGA-PRAD, which has information on gene expression in 497 primary PCa tumor samples compared with normal adjacent to tumor tissue.

Although the comparisons made in TCGA-PRAD and Oncomine were not exactly on the same tissues, a similarity could be observed between the expression profiles obtained in both analyses for most of the genes studied. Likewise, we evaluated the protein expression of these genes using The Human Protein Atlas database, in tumor and normal tissue samples. The protein profiles observed in this platform for most of the HO-1 interactors correlated with those already observed at the mRNA level in the datasets analyzed in Oncomine and TCGA-PRAD, thus evidencing that the expression profile of HO-1 interactors in PCa is dysregulated at the transcriptomic and protein levels.

Our next aim was to evaluate the risk of relapse associated with the expression of the genes of interest in PCa patients who had undergone radical prostatectomy. Results showed that upregulation of *RCC1* and *PDCD5* and downregulation of *ASPH*, *HSPB1*, *SQSTM1*, *ANXA2*, *GSN*, *PURA*, and *MX1*, comparing PCa vs. normal prostate or normal adjacent to tumor tissue, correlated with a decrease in relapse-free survival of PCa patients, revealing the clinical importance of the HO-1 interactors classified into Groups A or B.

In order to assess whether HO-1 was capable of modulating its interactors at the transcriptional level, we evaluated the transcriptomic profile of PC3 cells under genetic induction of HO-1 by RNA-seq. We paid special attention to MX1, since HO-1 modulation was capable of inducing MX1 expression. Altogether, the evidence obtained from the different analyses performed led us to focus on *MX1*, since: (1) it showed a significant over-expression under HO-1 induction; (2) it was under-expressed in PCa vs. normal prostate; and (3) its low expression was associated with a significant decrease in RFS for PCa patients. Further, there was a significant positive correlation between *MX1* and *HMOX1* expression in PCa patients.

Little is known about *MX1* and its association with tumorigenesis. Mushinski et al. [31] explored the molecular mechanisms of PCa metastasis using PC3 and PC3M (derived from a liver metastasis from a nude mouse bearing a splenic explant of PC3) cell lines. Results showed that *MX1* was expressed in PC3 and not in PC3M. Further, it was demonstrated that *MX1* transcription was inducible by type I, II, and III IFNs [32,33,34]. Both, type I and II IFNs have been used in the treatment of various types of cancer, including melanoma, follicular lymphoma, hairy cell leukemia, chronic myeloid leukemia, Kaposi’s sarcoma, and renal cell carcinoma, but the mechanisms of their anti-tumoral activity have not been fully deciphered [35]. Direct anti-proliferative effects on the tumor and indirect immunomodulatory effects on the host have been reported for type I and II IFNs. Shou et al. [36], Nagano et al. [33], and Schulz et al. [37] demonstrated that a significant proportion of genes whose downregulation is associated with PCa tumorigenesis were IFN-inducible genes, including *MX1*. However, MX1 has been mostly studied for its antiviral properties [14,15,16]. To test the hypothesis that MX1 played a role in reducing the motility and metastasis of PCa and other cancers, Mushinski et al. [31] over-expressed *MX1* in PC3M and LOX melanoma cells. *MX1* over-expression induced a clear reduction in motility and invasiveness in both cell lines compared with controls. Similarly, in vivo assays in SCID mice showed a significant reduction of liver metastases after intra-splenic injection of PC3M cells expressing *MX1* [31].

As shown in this study, genetic or pharmacological induction of HO-1 was able to induce *MX1* expression. Moreover, immunofluorescence assays revealed a significant increase in MX1 intensity under HO-1 induction. Interestingly, increased co-localization of HO-1 and MX1 was observed when cells over-expressed HO-1 compared with controls. Taking these results into account, we evaluated whether there was any correlation between these two genes in PCa patient tissues. The results evidenced a significant positive correlation between *MX1* and *HMOX1* expression profiles, both in human tumor and normal adjacent to tumor tissue samples. No correlation was observed in normal tissue samples, which highlights a potential association between both factors in prostate tumor tissues.

In 2008, Tomlins et al. first reported that approximately 50% of PCa cohorts had a fusion between *TMPRSS2* and *ERG* on chromosome 21 [38], which correlated with an invasive phenotype and was frequently associated with interstitial DNA deletions spanning the *MX1* locus [39]. These deletions are associated with an increase in PCa aggressiveness. In this work, we also analyzed whether there were genetic alterations in *MX1* associated with PCa and whether these alterations were related to the decreased expression profile seen in the disease. The cBioPortal platform, which included studies of RNA-seq and copy-number alterations for PCa patient samples (*n* = 3211), showed that deletion was the most frequent genetic alteration in *MX1*. Subsequently, we analyzed whether there was a correlation between *MX1* copy number alterations and its expression profile. Results evidenced that samples with a complete deletion for *MX1* displayed decreased mRNA expression. The same analysis was carried out for *HMOX1*, in which deletions were rarely observed throughout the samples, without it being associated with decreased *HMOX1* mRNA levels.

As mentioned before, *MX1* plays an important role in anti-viral activity [14,15,16]. Previous literature reported the association between viral infection and regulation of ERS [40]. Numajiri et al. demonstrated that *MX1* potentiates ERS signaling and ERS-induced cell death after Swiss 3T3 cells (cell line derived from mouse embryo fibroblasts) were infected with the Influenza virus [41]. Furthermore, MX1 induction increased *HSPA5* (BiP) and *DDIT3* (CHOP) mRNA levels and *XBP1* alternative splicing, leading to the expression of genes involved in endoplasmic reticulum degradation and cell death [41]. Therefore, MX1 may function as an accelerator of ERS-mediated apoptosis.

Hence, we set out to analyze the role of *MX1* in ERS, UPR pathways, and ERS-associated apoptosis in PCa. UPR activation is considered the criterion that defines ERS [10]. This mechanism aims to safeguard cell survival against injury or initiate cell apoptosis in cases of excessive stress. To address this goal, we generated ERS using thapsigargin, which was confirmed by a significant induction of *HSPA5*, *DDIT3*, and *XBP1*. *MX1* was also significantly induced under ERS, and strikingly, *MX1* silencing was able to decrease thapsigargin-induced ERS significantly, mainly evidenced by *HSPA5* downregulation. Further, we evaluated the effect of ERS on apoptosis and autophagy. Results showed that, under ERS stimulus, apoptosis and autophagy significantly increased in PC3 cells.

Considering that *HMOX1* was able to modulate *MX1*, we proposed to study whether the alteration of *MX1*, *HMOX1*, and ERS genes’ expression could alter the biochemical RFS of PCa patients. Results showed that an increase in *MX1*, *HMOX1*, *DDIT3*, and *HSPA5* expression reduced the risk of biochemical relapse in PCa patients who had undergone radical prostatectomy. Additionally, patients with a higher risk of relapse due to low *MX1* expression, had a better prognosis with higher *HMOX1* levels. This work is the first to associate *HMOX1*, *MX1*, and ERS in PCa.

The toxicity caused by the accumulation of misfolded proteins can be so severe for the cell that UPR has evolved as the main mechanism to deal with this threat, and depending on the intensity and duration of stress, UPR can tip the balance towards cell survival or death [10]. When cells experience a mild to moderate stress signal with a short duration, UPR activates the pro-survival module that seeks to neutralize the initial stress so that cells can adapt to it. Conversely, if the stress signal is intense and/or lasts over time, the UPR activates the pro-death module, which aims to lead the cell to apoptosis [26].

**Figure 6:**
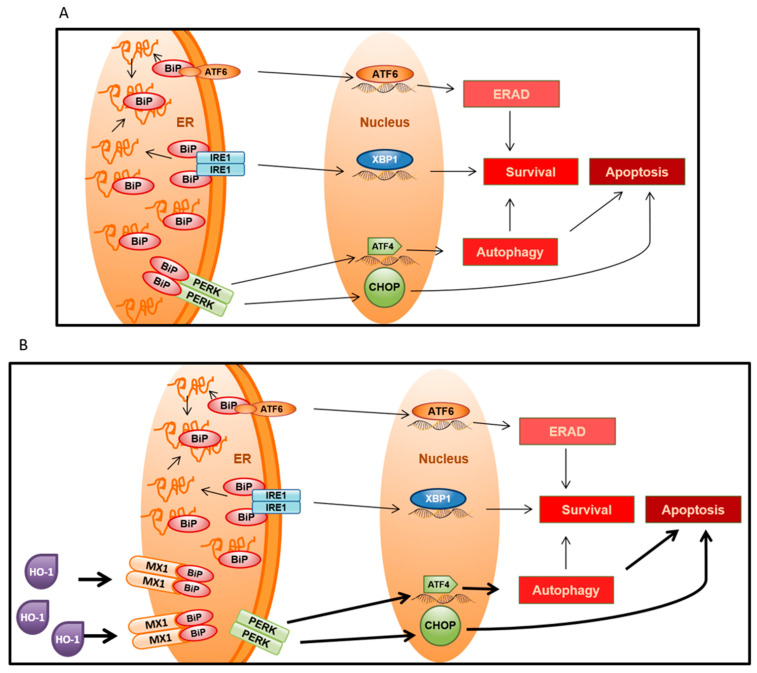
Schematic representation of the proposed model for MX1’s role in ERS-mediated cell fate. (**A**) When misfolded proteins begin to accumulate within the ER lumen, the BiP chaperone breaks free from PERK, IRE1, and ATF6 to bind to misfolded proteins, activating the unfolded protein response (UPR). The intensity and duration of ERS are decisive factors that define the cellular fate to survival or death [10]. (**B**) MX1 may sequester BiP, enhancing PERK-mediated pathway activation, leading to cell death. Further, HO-1 may favor this ERS axis, enhancing MX1 levels. ERAD = Endoplasmic-reticulum-associated protein degradation.

The pro-survival module is well represented by BiP [10], which is encoded by *HSPA5*. The pro-death module is represented by CHOP, encoded by *DDIT3* (Figure 6A). In the absence of stress, the BiP chaperone is associated with transmembrane sensors: Endoplasmic reticulum pancreatic kinase (PERK), enzyme 1 with inositol requirement (IRE1), and factor 6 transcription activator (ATF6). The UPR mechanism starts when the BiP chaperone is sequestered by the misfolded proteins that begin to accumulate in the reticular lumen [26]. This event causes the release of the transmembrane sensors IRE1, ATF6, and PERK from the ER, triggering the activation of UPR, ultimately determining cell fate (Figure 6A).

Reports have evidenced that: (1) MX1 improves ERS signaling via CHOP induction and promotes ERS-mediated apoptosis [41]; (2) the C-terminal region of MX1 is critical for interaction with BiP [42]; and (3) our results showed that *MX1* silencing directly decreases *DDIT3* expression; therefore, we may suggest that MX1 over-expression causes BiP sequestering, enhancing PERK-mediated pathway activation, leading to cell death (Figure 6B). Further, HO-1 may favor this ERS axis, enhancing MX1 levels, tilting the balance to pro-death events in PCa.

## 5. Conclusions

The results of this study evidenced that MX1 could drive the ERS balance towards pro-death events in malignant prostatic pathology, making it a promising target for the development of new therapies for PCa. Therefore, generating drugs that could induce MX1 might be a promising therapeutic tool to provide more specific antitumor activity and less toxicity than other treatments. In this sense, HO-1 induction by hemin, an FDA-approved drug, could be an alternative route to boost MX1.

## Figures and Tables

**Table 1:** Meta-analysis of multiple microarray datasets for HO-1-interacting proteins. Expression microarray studies selected from the Oncomine platform (http://www.oncomine.org) comparing prostate adenocarcinoma vs. normal prostate. N/A = not available.

N°	Dataset	Platform	Measured Genes	N° Samples	GEO Accession
1	Arreduani Prostate	HG U133 Plus 2.0 Array	19,574	21	GSE55945
2	Grasso Prostate	Agilent Human Genome 44K	19,189	122	GSE35988
3	Holzbeierlein Prostate	HG U95A-Av2 Array	8603	54	N/A
4	Lapointe Prostate	Undefined Platform	10,166	112	GSE3933
5	LaTulippe Prostate	HG U95A-Av2 Array	8603	35	GSE68882
6	Liu Prostate	HG U133A Array	12,624	57	N/A
7	Luo Prostate	Hu35k (A-D) and HG U95A-Av2 array	15,302	30	GSE68545
8	Magee Prostate	HumanGeneFL Array	5338	15	N/A
9	Singh Prostate	HG U95A-Av2 Array	8603	102	GSE68907
10	Taylor Prostate	Undefined Platform	22,238	185	GSE21032
11	Tomlins Prostate	Undefined Platform	10,656	101	GSE6099
12	Vanaja Prostate	HG U133(A-B) Array	17,779	40	N/A
13	Varambally Prostate	HG U133 Plus 2.0 Array	19,574	19	GSE3325
14	Wallace Prostate	HG U133 2.0 Array	12,603	89	GSE6956
15	Welsh Prostate	HG U95A-Av2 Array	8603	34	N/A
16	Yu Prostate	HG U95A-Av2 Array	8603	112	GSE68555

**Table 2:** General comparative summary of gene expression and the effects on relapse-free survival (RFS). The table summarizes the global analysis of the HO-1 interactome genes corresponding to Clusters A and B using Oncomine (16 datasets, *n* = 1045) and TCGA-PRAD (*n* = 497). The arrows indicate over- (red) or under-expression (blue) when comparing PCa vs. normal or normal adjacent to tumor tissue. Meta-analyses determined a median rank (position) for each gene assessed across all datasets. RFS associated with the gene expression profiles of Clusters A and B are shown for the Ross–Adams dataset (GSE70770) (*n* = 206). All comparisons considered low expression patients as the reference group. HR: hazards ratio. N/A: not available. Genes selected for further analysis are highlighted with * next to their gene symbol.

		EXPRESSION						SURVIVAL	
		Oncomine			TCGA			Ross Adams	
Expression Cluster	Gene	Over- or Under-Expression	Median Rank	*p*-Value	Over- or Under-Expression	Fold Change	*p*-Value	Cox *p*- Value	Hazards Ratio (IC95)
A	CBX3	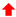	825 (top 20%)	4.21 × 10^−04^	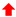	1.312	8.67 × 10^−12^	0.085	0.65 (0.4–1.06)
	RCC1 *	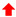	487 (top 15%)	0.012	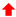	1.74	6.48 × 10^−31^	0.034	4.85 (1.20–2.09)
	TMOD3	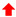	2035 (top 22%)	0.016	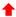	1.152	9.23 × 10^−05^	0.001	0.42 (0.26–0.69)
	NOA1	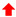	203 (top 3%)	3.76 × 10^−06^	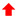	1.762	9.53 × 10^−40^	…	…
	EEF2	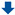	299 (top 24%)	1.06 × 10^−05^	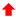	2.43	1.31 × 10^−73^	0.0002	0.30 (0.15–0.59)
	ASPH *	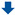	798 (top 9%)	0.003	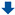	0.613	1.87 × 10^−13^	0.001	0.34 (0.18–0.66)
	HSPB1*	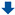	182.5 (top 11%)	1.06 × 10^−04^	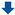	0.584	9.95 × 10^−24^	0.001	0.36 (0.20–0.66)
	SQSTM1 *	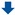	1581 (top 35%)	0.003	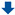	0.961	9.39× 10^−01^	0.009	0.40 (0.2–0.79)
	ANXA2 *	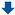	133 (top 16%)	0.003	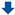	0.496	4.48 × 10^−19^	0.001	0.40 (0.23–0.70)
	GSN *	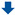	586.5 (top 10%)	0.005	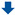	0.533	3.4 × 10^−21^	0.001	0.27 (0.12–0.60)
B	TOP1	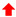	1082 (top 25%)	0.003	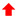	1.468	5.58 × 10^−14^	0.005	0.48 (0.29–0.80)
	PDCD5 *	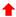	836.5 (top 22%)	0.011	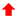	1.164	2.41 × 10^−05^	0.0001	5.80 (2.72–12.32)
	THRAP3	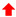	1898 (top 24%)	0.002	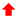	1.073	5.84 × 10^−05^	0.004	0.49 (0.3–0.8)
	SLC12A7	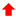	1914 (top 25%)	5.5 × 10^−04^	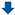	0.849	3.86 × 10^−01^	0.001	0.39(0.19–0.65)
	PURA *	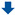	1280 (top 16%)	0.031	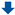	0.951	1.87 × 10^−01^	0.001	0.41 (0.25–0.69)
	RPA1	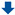	2093 (top 23%)	7.71× 10^−04^	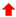	1.246	5.39 × 10^−06^	0.013	2 (1.16–3.47)
	SFSR3	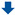	793 (top 11%)	0.01	N/A	…	…	0.024	1.77 (1.08–2.91)
	MX1 *	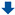	1542 (top 19%)	0.031	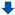	0.578	1.04 × 10^−07^	0.0044	0.47 (0.28–0.80)

## Supplementary Materials

The following are available online at https://www.mdpi.com/2218-273X/10/7/1005/s1, Figure S1: Analysis of HO-1 interactors as risk predictors of clinical outcome, Figure S2: Modulation of *MX1* and ERS genes expression by IFNγ and hemin.

## References

[1] Dulak J., Jozkowicz A. (2014). Novel Faces of Heme Oxygenase-1: Mechanisms and Therapeutic Potentials. Antioxidants Redox Signal..

[2] Chau L.-Y. (2015). Heme oxygenase-1: Emerging target of cancer therapy. J. Biomed. Sci..

[3] Nitti M., Piras S., Marinari U.M., Moretta L., Pronzato M.A., Furfaro A.L. (2017). HO-1 Induction in Cancer Progression: A Matter of Cell Adaptation. Antioxidants.

[4] Gueron G., De Siervi A., Ferrando M., Salierno M., De Luca P., Elguero B., Meiss R., Navone N., Vazquez E. (2009). Critical Role of Endogenous Heme Oxygenase 1 as a Tuner of the Invasive Potential of Prostate Cancer Cells. Mol. Cancer Res..

[5] Ferrando M., Gueron G., Elguero B., Giudice J., Salles A., Leskow F.C., Jares-Erijman E.A., Colombo L., Meiss R., Navone N. (2011). Heme oxygenase 1 (HO-1) challenges the angiogenic switch in prostate cancer. Angiogenesis.

[6] Gueron G., Giudice J., Valacco P., Paez A., Elguero B., Toscani M., Jaworski F., Leskow F.C., Cotignola J., Martí M.A. (2014). Heme-oxygenase-1 implications in cell morphology and the adhesive behavior of prostate cancer cells. Oncotarget.

[7] Paez A., Vazquez E., Gueron G. (2017). Heme oxygenase 1 governs the cytoskeleton at filopodia: Pulling the brakes on the migratory capacity of prostate tumoral cells. Cell Death Discov..

[8] Paez A.V., Pallavicini C., Schuster F., Valacco M.P., Giudice J., Ortiz E.G., Anselmino N., Labanca E., Binaghi M., Salierno M. (2016). Heme oxygenase-1 in the forefront of a multi-molecular network that governs cell-cell contacts and filopodia-induced zippering in prostate cancer. Cell Death Dis..

[9] Łoboda A., Damulewicz M., Pyza E., Jozkowicz A., Dulak J. (2016). Role of Nrf2/HO-1 system in development, oxidative stress response and diseases: An evolutionarily conserved mechanism. Cell. Mol. Life Sci..

[10] Schönthal A.H. (2012). Endoplasmic Reticulum Stress: Its Role in Disease and Novel Prospects for Therapy. Scientifica.

[11] Malhotra J.D., Kaufman R.J. (2007). Endoplasmic Reticulum Stress and Oxidative Stress: A Vicious Cycle or a Double-Edged Sword?. Antioxidants Redox Signal..

[12] McCullough K.D., Martindale J.L., Klotz L.-O., Aw T.-Y., Holbrook N.J. (2001). Gadd153 Sensitizes Cells to Endoplasmic Reticulum Stress by Down-Regulating Bcl2 and Perturbing the Cellular Redox State. Mol. Cell. Boil..

[13] Rutkowski D.T., Arnold S.M., Miller C.N., Wu J., Li J., Gunnison K.M., Mori K., Akha A.A.S., Raden D., Kaufman R.J. (2006). Adaptation to ER Stress Is Mediated by Differential Stabilities of Pro-Survival and Pro-Apoptotic mRNAs and Proteins. PLoS Boil..

[14] Pavlovic J., Zürcher T., Haller O., Staeheli P. (1990). Resistance to influenza virus and vesicular stomatitis virus conferred by expression of human MxA protein. J. Virol..

[15] Kochs G., Janzen C., Hohenberg H., Haller O. (2002). Antivirally active MxA protein sequesters La Crosse virus nucleocapsid protein into perinuclear complexes. Proc. Natl. Acad. Sci. USA.

[16] Haller O., Staeheli P., Schwemmle M., Kochs G. (2015). Mx GTPases: Dynamin-like antiviral machines of innate immunity. Trends Microbiol..

[17] Livak K.J., Schmittgen T.D. (2001). Analysis of relative gene expression data using real-time quantitative PCR and the 2(-Delta Delta C(T)) method. Methods.

[18] Anselmino N., Bizzotto J., Sanchis P., Lage-Vickers S., Ortiz E., Valacco P., Paez A., Labanca E., Meiss R., Navone N. (2020). HO-1 Interactors Involved in the Colonization of the Bone Niche: Role of ANXA2 in Prostate Cancer Progression. Biomolecules.

[19] Ross-Adams H., Lamb A.D., Dunning M., Halim S., Lindberg J., Massie C.E., Egevad L., Russell R., Ramos-Montoya A., Vowler S. (2015). Integration of copy number and transcriptomics provides risk stratification in prostate cancer: A discovery and validation cohort study. EBioMedicine.

[20] Kassambara A., Kosinski M., Biecek P. survminer: Drawing Survival Curves using “ggplot2” 2019. Computer program.

[21] Budczies J., Klauschen F., Sinn B.V., Győrffy B., Schmitt W., Darb-Esfahani S., Denkert C. (2012). Cutoff Finder: A Comprehensive and Straightforward Web Application Enabling Rapid Biomarker Cutoff Optimization. PLoS ONE.

[22] Bussolati B., Ahmed A., Pemberton H., Landis R.C., Di Carlo F., Haskard D., Mason J.C. (2004). Bifunctional role for VEGF-induced heme oxygenase-1 in vivo: Induction of angiogenesis and inhibition of leukocytic infiltration. Blood.

[23] Wegiel B., Chin B.Y., Otterbein L.E. (2008). Inhale to survive, cycle or die? Carbon monoxide and cellular proliferation. Cell Cycle.

[24] Weinstein J.N., Li J., Mills G.B., Shaw K.R.M., Ozenberger B.A., Ellrott K., Shmulevich I., Sander C., Stuart J.M., The Cancer Genome Atlas Research Network (2013). The Cancer Genome Atlas Pan-Cancer analysis project. Nat. Genet..

[25] Wang M., Kaufman R.J. (2014). The impact of the endoplasmic reticulum protein-folding environment on cancer development. Nat. Rev. Cancer.

[26] Verfaillie T., Garg A.D., Agostinis P. (2013). Targeting ER stress induced apoptosis and inflammation in cancer. Cancer Lett..

[27] Jozkowicz A., Was H., Dulak J. (2007). Heme Oxygenase-1 in Tumors: Is It a False Friend?. Antioxidants Redox Signal..

[28] Saccà P., Meiss R., Casas G., Mazza O., Calvo J.C., Navone N., Vazquez E. (2007). Nuclear translocation of haeme oxygenase-1 is associated to prostate cancer. Br. J. Cancer.

[29] Jaworski F.M., Gentilini L., Gueron G., Meiss R.P., Ortiz E.G., Berguer P.M., Ahmed A., Navone N., Rabinovich G.A., Compagno D. (2017). In Vivo Hemin Conditioning Targets the Vascular and Immunologic Compartments and Restrains Prostate Tumor Development. Clin. Cancer Res..

[30] Elguero B., Gueron G., Giudice J., A Toscani M., De Luca P., Zalazar F., Leskow F.C., Meiss R., Navone N., De Siervi A. (2012). Unveiling the Association of STAT3 and HO-1 in Prostate Cancer: Role beyond Heme Degradation. Neoplasia.

[31] Mushinski J.F., Nguyen P., Stevens L.M., Khanna C., Lee S., Chung E.J., Lee M.-J., Kim Y.S., Linehan W.M., Horisberger M.A. (2009). Inhibition of Tumor Cell Motility by the Interferon-inducible GTPase MxA. J. Boil. Chem..

[32] A Horisberger M. (1992). Interferon-induced human protein MxA is a GTPase which binds transiently to cellular proteins. J. Virol..

[33] Nagano K., Masters J.R., Akpan A., Yang A., Corless S., Wood C., Hastie C., Zvelebil M., Cramer R., Naaby-Hansen S. (2003). Differential protein synthesis and expression levels in normal and neoplastic human prostate cells and their regulation by type I and II interferons. Oncogene.

[34] Kotenko S.V., Gallagher G., Baurin V.V., Lewis-Antes A., Shen M., Shah N.K., Langer J., Sheikh F., Dickensheets H., Donnelly R.P. (2002). IFN-λs mediate antiviral protection through a distinct class II cytokine receptor complex. Nat. Immunol..

[35] Jonasch E., Haluska F.G. (2001). Interferon in Oncological Practice: Review of Interferon Biology, Clinical Applications, and Toxicities. Oncologist.

[36] Shou J., Soriano R., Hayward S.W., Cunha G.R., Williams P.M., Gao W.-Q. (2002). Expression profiling of a human cell line model of prostatic cancer reveals a direct involvement of interferon signaling in prostate tumor progression. Proc. Natl. Acad. Sci. USA.

[37] Schulz W.A., Alexa A., Jung V., Hader C., Hoffmann M.J., Yamanaka M., Fritzsche S., Wlazlinski A., Müller M., Lengauer T. (2007). Factor interaction analysis for chromosome 8 and DNA methylation alterations highlights innate immune response suppression and cytoskeletal changes in prostate cancer. Mol. Cancer.

[38] Tomlins S., Laxman B., Varambally S., Cao X., Yu J., Helgeson B.E., Cao Q., Prensner J.R., Rubin M.A., Shah R.B. (2008). Role of the TMPRSS2-ERG Gene Fusion in Prostate Cancer. Neoplasia.

[39] Attard G., Clark J., Ambroisine L., Fisher G., Kovacs G., Flohr P., Berney D.M., Foster C.S., Fletcher A., Gerald W.L. (2007). Duplication of the fusion of TMPRSS2 to ERG sequences identifies fatal human prostate cancer. Oncogene.

[40] Isler J.A., Skalet A.H., Alwine J.C. (2005). Human Cytomegalovirus Infection Activates and Regulates the Unfolded Protein Response. J. Virol..

[41] Haruki A.N., Naito T., Nishie T., Saito S., Nagata K. (2011). Interferon-Inducible Antiviral Protein MxA Enhances Cell Death Triggered by Endoplasmic Reticulum Stress. J. Interf. Cytokine Res..

[42] Medigeshi G.R., Lancaster A.M., Hirsch A.J., Briese T., Lipkin W.I., DeFilippis V., Früh K., Mason P.W., Nikolich-Žugich J., Nelson J.A. (2007). West Nile Virus Infection Activates the Unfolded Protein Response, Leading to CHOP Induction and Apoptosis. J. Virol..

