# Supplementary material for: Myxovirus Resistance Protein 1 (MX1), a Novel HO-1 Interactor, Tilts the Balance of Endoplasmic Reticulum Stress towards Pro-Death Events in Prostate Cancer
Source: Biomolecules. 2020 Jul 6;10(7):1005. doi: 10.3390/biom10071005 (PMC7407234; doi:10.3390/biom10071005)
Supplement: Supplementary file 1 [file biomolecules-10-01005-s001.pdf]

Supplementary figure 1

A

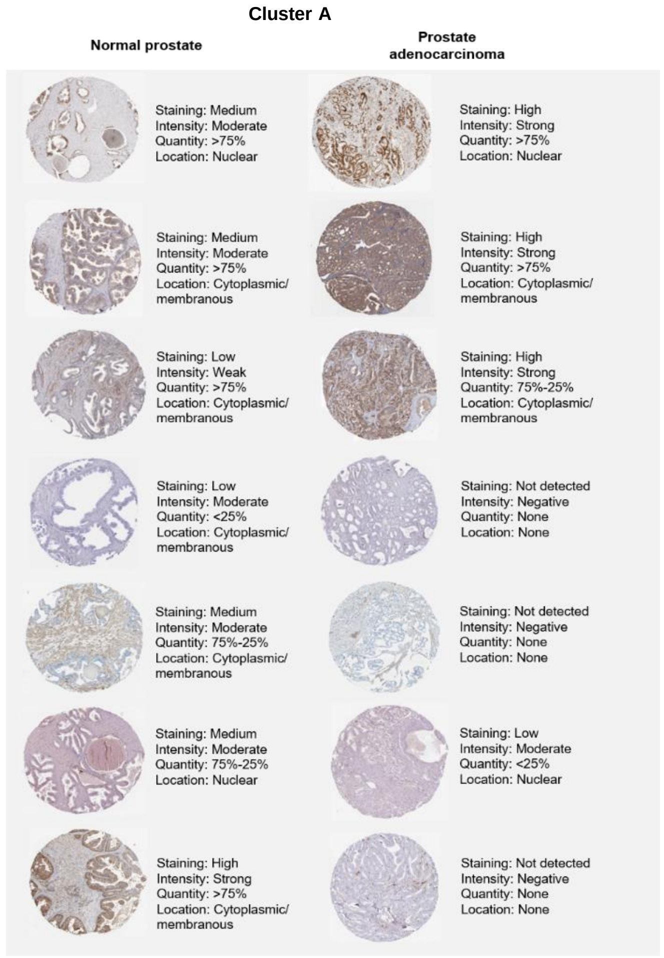

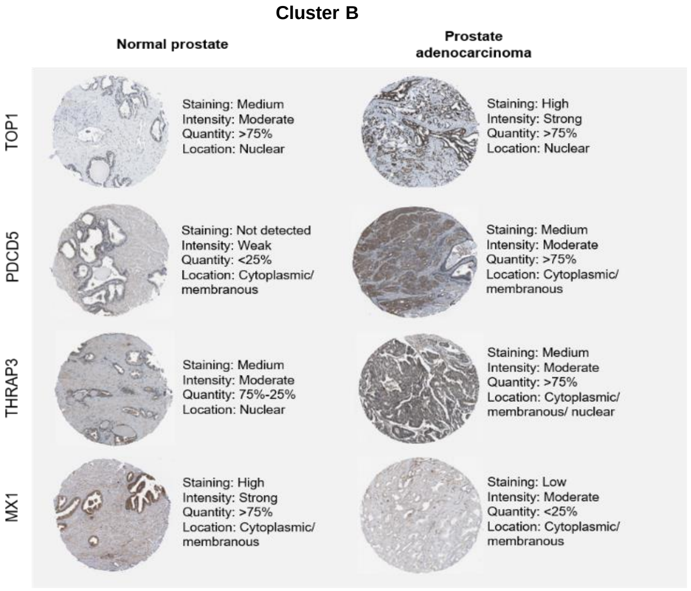

Supplementary Figure 1. Analysis of HO-1 interactors as risk predictors of clinical outcome. A) Protein expression for Cluster A and B genes whose expression correlated with mRNA expression profile, measured by immunohistochemistry in PCa samples from The Human Protein Atlas. B) Kaplan-Meier (KM) curves for relapse free survival (RFS) in months for PCa patients with low (blue) or high (red) expression of Cluster A and B in the Ross Adams dataset (GSE70770) (n= 206). All comparison considered low expression patients as the reference group. In all cases, the logrank, Hazard Ratio (HR) and Cox P values obtained for each curve are indicated.

B

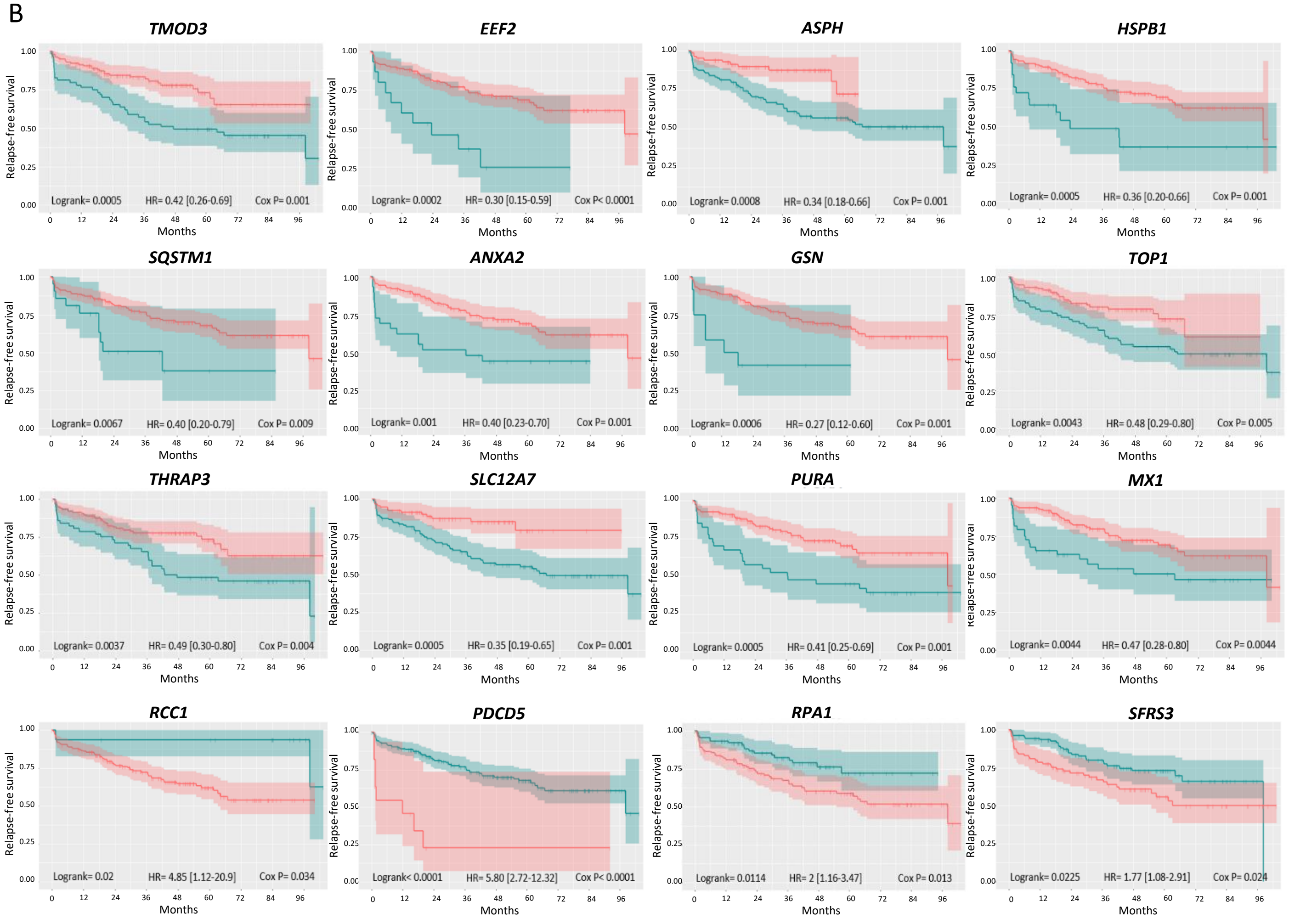

Supplementary figure 2

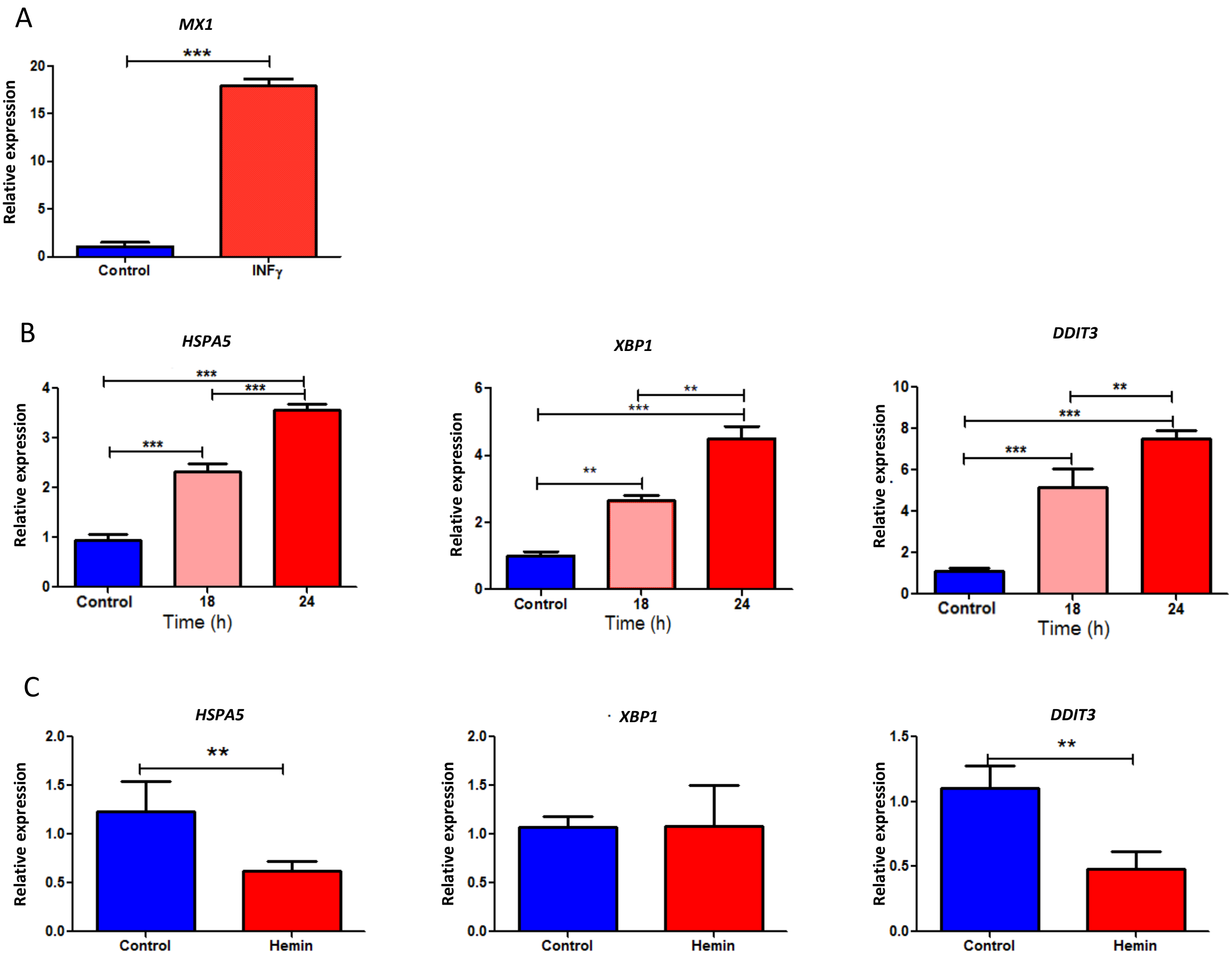

Supplementary Figure 2. Modulation of MX1 and ERS genes expression by INFγ and hemin. A) *MX1* expression assessed by RT-qPCR in PC3 cells treated with INFγ (500U / ml; 18h) or PBS as control. B) *HSPA5*, *XBP1* and *DDIT3* expressions were assessed by RT-qPCR in PC3 cells treated or not with INFγ (500U / ml; 18 and 24 h) or C) treated with hemin (80 μM, 24 h). PBS was used as control treatment. Values were relativized to *PPIA* as a reference gene and normalized to the control condition. One representative of at least three independent experiments is shown. Results are shown as mean ± s.d. Statistical significance: * $p < 0.05$; ** $p < 0.01$; *** $p < 0.001$.
